# New pandemics: HIV and AIDS, HCV and chronic hepatitis, Influenza virus and flu

**DOI:** 10.1186/1742-4690-4-8

**Published:** 2007-02-01

**Authors:** Anne Gatignol, Jean Dubuisson, Mark A Wainberg, Éric A Cohen, Jean-Luc Darlix

**Affiliations:** 1 Lady Davis Institute for Medical Research, and Department of Medicine, Microbiology and Immunology, McGill University, Montréal, Québec, Canada; 2 Institut de Biologie de Lille (UMR8161), CNRS, Université de Lille I & II and Institut Pasteur de Lille, Lille, France; 3 McGill University AIDS Centre, Montréal, Québec, Canada; 4 Institut de Recherches Cliniques de Montréal, Department of Microbiology and Immunology, Université de Montréal, Québec, Canada; 5 LaboRetro, Unité INSERM 758 de Virologie Humaine, École Normale Supérieure de Lyon, Lyon, France

## Abstract

New pandemics are a serious threat to the health of the entire world. They are essentially of viral origin and spread at large speed. A meeting on this topic was held in Lyon, France, within the XIXth Jacques Cartier Symposia, a series of France-Québec meetings held every year. New findings on HIV and AIDS, on HCV and chronic hepatitis, and an update on influenza virus and flu were covered during this meeting on December 4 and 5, 2006. Aspects of viral structure, virus-host interactions, antiviral defenses, drugs and vaccinations, and epidemiological aspects were discussed for HIV and HCV. Old and recent data on the flu epidemics ended this meeting.

## Background

The XIXth Jacques Cartier Symposium was held to discuss recent advances on new viral pathogens that are rapidly expanding such as the human immunodeficiency virus (HIV-1) causing AIDS, the hepatitis C virus (HCV), causing chronic hepatitis and hepatocellular carcinoma, and influenza virus causing flu. These viral infections all have important human, social and political consequences worldwide. The opening lecture by the director of the French national agency against AIDS and hepatitis was followed by a session on the structural biology of HIV and HCV and the biochemistry of essential viral components. In the second session, aspects of virus-host relationships were discussed and the third one tackled problems of innate immunity, anti-viral defenses and counteractions. Session 4 dealt with anti-viral drugs and vaccination and session 5 evoked epidemiological aspects of HIV and HCV transmission. Finally, an update on the previous and pending flu epidemics ended the meeting.

In his opening lecture **Jean-François Delfraissy**, the new director of the Agence Nationale de Recherches sur le SIDA et les hépatites (ANRS, Paris, France), gave a brief outline of the major tasks of ANRS, namely the support of fundamental research on HIV and HCV/HBV, based on excellency. JF Delfraissy also summarized the agency major efforts in the growing field of anti-HIV vaccination and prevention including education, training and circumcision. He cited ongoing and published studies on effectiveness and safety of genotyping, the ART-LINC cohort the TRIVACAN assay, HIV and tuberculosis ongoing with the National Institutes of Health, USA, the circumcision study, coinfection with HSV2, HIV vaccination of HIV-infected individuals, and therapeutic assays on HIV/HCV coinfection [1-5].

### Structural biology and biochemistry of HIV and HCV

In this session, **Elena Chertova **(Frederick NCI/NIH, USA) presented outstanding data on the characterization of trimeric envelope structures on the surface of HIV-1 and SIV virions. Biochemical analyses, electron tomography and image processing support an overall virion structure where about 8–9 ENV trimers are found on a HIV-1 virion and 70–79 on the average on mutant SIV known to contain a high level of the viral envelope [6-8]. These ENV trimer counts combined with biochemical analyses allow calculation of the number of gag molecules per virion, yielding a value of approximately 1400. These results demonstrate the presence of envelope trimers on the virion surface, and have important implications for understanding virion formation, virus-cell interactions, and virus neutralization.

Because the genome of HIV, HCV and influenzae viruses is made of RNA, the talk given by **Renée Schröeder **(University of Vienna, Austria) addressed the major issue on how RNA molecules can adopt a functionally relevant conformation, amongst billions of possible structures, necessary for their expression and their replication. A number of RNA cofactors have been discovered, which include specific nucleic acids binding proteins, chaperones and helicases [9-11]. In that respect, RNA chaperones appear to be wide spread in nature, where they help RNA molecules rapidly reach their functional conformation, in physiological conditions and in the absence of ATP.

To pursue on this theme of viral RNA conformation, **Jean-Luc Darlix **(INSERM, École Normale Supérieure, Lyon, France) summarized a decade of research dedicated to the nucleocapsid protein NC of HIV-1. In fact, NC is both a specific nucleic acid binding protein and a potent RNA chaperone that guides reverse transcriptase (RT) during proviral DNA synthesis, and selects and dimerizes the genomic RNA during virus assembly (see also talk of Delphine Muriaux) [12-15]. JL Darlix also presented recent data showing that NC can control the level of nucleotide misincorporation during cDNA synthesis by RT, via specific NC-RT-cDNA interactions allowing RT-mediated nucleotide excision-repair.

To continue on the NC protein of HIV-1, **Yves Mély **(CNRS, Faculté de Pharmacie, Strasbourg) gave an overview on the 3D structure of NC highlighting the fact that this small viral protein, formed of two zinc fingers flanked by basic residues, operates through an hydrophobic plateau. Mutating this plateau results in the production of non-infectious viruses. A chemical class of anti-NC agents have been selected that specifically interact with the NC plateau. As illustrated by Y. Mély, some of these agents were found to completely inhibit the chaperoning function of NC [16,17].

**HCV **has come a long way since its discovery in 1989 with the recent establishement of a cell culture system to investigate its replication and biogenesis [18]. In the mean time, several surrogate models have been developed to study some steps of HCV life cycle. Virus-like particles (VLPs) obtained by expressing the genes encoding HCV structural proteins in mammalian cells have been used as a model for investigating HCV morphogenesis. **Philippe Roingeard **(INSERM, Université de Tours, France) reported recent data on the morphogenesis of HCV VLPs at the level of the endoplasmic reticulum (ER) membrane, describing HCV core protein domains required or dispensable for this phenomenon [19]. Results also indicate that the processing of the HCV core protein by the signal peptide peptidase is required for HCV VLP assembly [20].

As well documented for HIV-1, HCV also shows remarkable sequence variability due to the lack of a proofreading activity of the HCV RNA-dependent RNA polymerase. The HCV dedicated databases such as the European Hepatitis C Virus Database (euHCVdb) [21,22] allow the investigation of the genetic and structural variability of all available HCV sequences as presented by **François Penin **(CNRS, IBCP, Lyon, France). Despite the high degree of variability, amino acid sequence analyses reveal a global conservation of protein structures. Interestingly, the conservation of some amino acids in membrane protein domains is likely related to their essential role in the formation of the RNA replication complex, which is associated with membranes [23]. The hydrophilic amino acids generally located at the protein surface are the most variable, offering a high potential to modulate interactions with host components, and thus a better replicative fitness of the variants.

### Virus-host relationships

Because viruses such as HIV, HCV and Influenzae are parasites, virus-cell interactions are required at all steps of the virus replication cycle. Specific molecular events between viral and cellular components mediate viral entry, trafficking, expression and release [24]. Some aspects of these virus-host relationships have been addressed. HIV-1 entry into the target cell involves the formation of a trimolecular complex consisting of SUgp120, a CD4 receptor and a chemokine co-receptor. **Michel J. Tremblay **(CHUL, Université Laval, Québec, Canada) discussed interactions between cell-derived components incorporated into virions and their natural counter-receptors for their contribution to HIV-1 entry/infection. He showed that the presence of host-derived ICAM-1 on virions results in a 5 to 10 fold increase in virus infectivity on target T-cells and nearly 100 folds when they infect activated T cells expressing LFA-1. Virus entry studies including subcellular fractionation experiments with CD4^+ ^T cells demonstrated that the acquisition of ICAM-1 by nascent HIV-1 modified the entry route of the virus, more likely to release their material within the cell cytosol instead of being endocytosed. Activation of CD4^+ ^T cells resulted in LFA-1 clustering, promoting the early events of HIV-1 replication through an interaction between virus-embedded host ICAM-1 and LFA-1 clusters. HIV-1 is concentrated in microdomains rich in LFA-1 clusters [25,26].

HIV-1 expression is controlled by the viral trans-activator Tat. Recently, RNA interference (RNAi) appeared to be a major pathway of gene regulation both by exogenous small interfering (si) RNAs and by endogenous viral or cellular micro (mi) RNAs. **Kuan-Teh Jeang **(NIAID, NIH, Bethesda, USA) spoke on several topics related to Tat and miRNAs. First, he presented new data addressing the possibility that Tat is a virion-associated protein since he has previously suggested that Tat might be in virions [27]. Next, he touched upon how HIV-1 infection, perhaps through Tat-Dicer interaction [28,29], alters the expression profile of miRNAs in human cells [30]. He commented on different miRNA signatures in PBMCs from groups of AIDS patients at various stages of disease progression. Finally, KT Jeang reported that the HIV-1 PBS-tRNA structure could be a Dicer-processed substrate in infected cells.

Viruses use the translation cellular machinery to synthesize their proteins. **Théophile Ohlmann **(INSERM-ENS, Lyon, France) showed that retroviruses have developed structural features and strategies that enable them to take over from the cellular translation machinery. Lentiviruses HIV-1, HIV-2, FIV and SIV have been shown to initiate translation by Internal Ribosome Entry Segments (IRES). These IRESes have been mapped in the 5'UTR and in the gag coding region and play an important role in the control of viral protein synthesis. He showed that IRES from HIV-2 is of a novel type. In HIV-2, 3 AUG start codons initiate the synthesis of 3 gag isoforms and leaderless translation is very efficient showing the role of the IRES. In addition the translation initiation factor eIF4G can be cleaved by HIV-1 and HIV-2 proteases, which modifies the AUG selection on its cognate RNA and has a different impact on both cellular and viral translation. Finally, the binding of the Gag polyprotein to the 5' leader has an important impact on the modulation of viral protein synthesis [31].

Viral translation and consequently viral replication is also influenced by cellular factors. **Anne Gatignol **(Lady Davis Institute, McGill University, Montréal, Canada) talked about the cellular response to HIV infection mediated by PKR and RNAi and its control by the cellular TAR RNA binding protein (TRBP). She showed that PKR is not activated in HIV-infected lymphocytes, but is activated in HIV-infected astrocytes that do not replicate the virus efficiently. This difference in cell response is due to the low amount of TRBP in astrocytes that cannot counteract PKR activation [32,33]. TRBP is a cellular protein that inhibits PKR activation directly and by controlling the activity of the PKR activator PACT. Evidence on the control of PACT by TRBP was presented. TRBP is part of the RNA-induced silencing complex (RISC) that mediates RNAi [34]. It has been suggested that RNAi could be part of the cellular response to viral infection in mammals like in plants and lower eukaryotes. However, TRBP being part of the RISC and favoring HIV replication raises the question of whether HIV diverts a cellular pathway or evades from antiviral immunity [35]. She discussed the effects of TRBP or Dicer inhibition on HIV production and the activity of HIV on RNAi.

#### HIV-1 assembly

The HIV-1 structural Gag polyprotein is responsible for orchestrating the assembly process and alone is sufficient for the production of viral particles. Numerous recent studies have shown that HIV-1 Gag assembly could take place at the plasma membrane (PM) or/and in late endosomal/multivesicular compartments (LE/MVB) depending on the cell type, thus raising the possibility that LE/MVB may represent early intermediates where HIV-1 assembly is initiated [36,37]. **Delphine Muriaux **(INSERM-École Normale Supérieure, Lyon, France) discussed the intracellular trafficking and assembly of Gag and the role of Gag-RNA interactions in these processes. Using immunofluorescence/FISH coupled to confocal microscopy, sub-cellular fractionation and RT-PCR techniques, she showed that in the case of wild-type HIV-1 virus, Gag-mediated assembly and budding occur both at the PM and on intracellular endosomal membranes [38]. In the case of NC mutants, in which NC-RNA interactions are impaired [39,40], she found that NC-mutated Gag displayed decreased particle release and strongly accumulated at the PM. On the basis of these results, she favors the view that HIV-1 can assemble both at the PM and on LE/MVB membranes with the requirement of NC-RNA interactions for Gag assembly and trafficking.

**Éric A. Cohen **(IRCM, Université de Montréal, Montréal, Canada) addressed similar issues and presented an analysis of Gag trafficking in 293T cells where Gag localizes both at the PM and LE/MVB at steady-state. Using an approach that combined pulse chase-labeling and subcellular fractionation, he provided evidence indicating that the PM is the primary site of productive HIV-1 particle assembly. The majority of Gag and mature virions detected in LE/MVB were shown to result from an internalization process from the PM towards MVB. Next, he touched on host cell factors that might regulate the steady-state accumulation of Gag and mature virions in different cell types. He reported that expression of MHC-II molecules (HLA-DR), which was previously shown to induce a relocation of Gag and mature particles to LE/MVB [41] enhanced Gag and mature particle internalization from the PM.

#### HCV biology

Recently, a cell-culture system that allows a relatively efficient amplification of HCV (HCVcc) has been reported. This system is based on the transfection of Huh-7 cells with genomic HCV RNA of the genotype 2a JFH1 strain cloned from an individual with fulminant hepatitis [18]. As presented by **Jean Dubuisson **(CNRS, Institut Pasteur, Lille, France), the HCVcc system allows for the first time the study of the complete viral life cycle. Surprisingly, the envelope glycoproteins are detected in the ER, whereas the core protein is exclusively found in association with lipid droplets in cells infected by HCVcc [42]. Another major tool in the study of HCV has been the development of retrovirus pseudotypes containing HCV envelope glycoproteins that have been called HCVpp [43]. The HCVpp and HCVcc systems begin to reveal some information on HCV determinants and cellular factors involved in virus entry. CD81 tetraspanin, scavenger receptor BI and Claudin-1 have been shown to be important cellular entry factors for HCV. In addition, some lipoproteins or apolipoproteins can modulate the entry process. Finally, some glycans present on HCV envelope glycoproteins have been shown to play a role in virus entry.

### Innate immunity, anti-viral defenses and counteractions

Virus entry induces a cell response to the infection. Aspects of the innate immunity and the role of dendritic cells in the adaptive immune response against HIV and HCV were discussed. The mechanisms whereby HCV evades the host's immune defenses and establishes persistent infection remain elusive. As reported by **John Hiscott **(Lady Davis Institute, McGill University, Montréal, Canada) HCV NS3-4A protease has been shown to interfere with double-stranded RNA signaling pathways. It disrupts the cellular RNA helicase retinoic acid-inducible gene I (RIG-I) pathway through proteolysis of a newly discovered essential adaptor protein of interferon regulatory factor-3 (IRF-3) activation [44-46]. Due to its recent simultaneous discovery by four different groups, this adaptor protein has received four different names: IPS-1, Cardif, VISA and MAVS. NS3-4A cleavage of MAVS/IPS-1/VISA/Cardif caused its relocation from the mitochondrial membrane to the cytosolic fraction. The IKK-related kinase IKKε that colocalizes with MAVS at the mitochodrial membrane is also disrupted by NS3/4A expression. These data provide the first link between mitochondrial function, development of innate antiviral response and HCV evasion mechanisms [47].

A natural innate resistance to HIV-1 occurs in old world monkeys. **Jeremy Luban **(Colombia University, USA; IRB, Bellinzona, Switzerland) reported the discovery of TRIM5α or TRIM-Cyp as cellular restriction factors that mediate this resistance [48,49]. TRIM5 specificity in owl monkeys is conferred by a C-terminal CypA domain that binds CA of HIV-1, SIV AGM and FIV, or by the CA-specific C-terminal SPRY domain in other particular primate species. TRIM5 may be thought of as a cytoplasmic receptor within the innate immune system which recognizes CA-specific determinants on the retroviral protein core [50]. Thus far, HIV-1 escape from TRIM-Cyp has not been observed.

Dendritic cells (DCs) capture pathogens like HIV or HCV virions and present antigens, activating an adaptive immune response. A fraction of the captured HIV virions is transmitted to CD4^+ ^lymphocytes by cell-to-cell transfer through the formation of virological synapses between infected and target cells. **Olivier Schwartz **(Institut Pasteur, Paris, France) demonstrated the importance of cell contacts for HIV-1 transmission by altering cell-to-cell contact by gentle shaking. Using an assay to assess the relative contributions of free and cell-associated virions, he reported that cell-to-cell transfer is the predominant mode of HIV spread [51]. He next documented how HIV-1 infected lymphocytes poorly conjugated with antigen-presenting cells and form abnormal immunological synapses. TCR and Lck accumulated in the recycling endosomes in HIV-1 infected lymphocytes, altering the endocytic and signaling networks at the immunological synapse and facilitating viral spread [52].

The mechanism whereby HCV evades the host's immune defenses and establishes persistent infection remain elusive. With the requirement of functional Toll-like receptor (TLR) signaling pathways for full DC activation and generation of CD4^+ ^T cell memory responses [53,54], the presentation of **Daniel Lamarre **(CHUM, Montréal, Canada) revived the concept that specific HCV-DC interactions exert an inhibitory pressure on innate responses [55], (Rodrigue-Gervais IG, Jouan L, Beaulé G, Sauvé D, Bruneau J, Willems B, Sékaly RP, Lamarre D, manuscript submitted). Analyses of dendritic cells (DC) isolated from the blood of HCV infected patients showed that the myeloid DC subset displays impaired expression of IL-12 and TNF-α but not IL-6 or CCL3 in response to poly(I):poly(C) (TLR3 ligand) and LPS (TLR4 ligand). In addition, attenuation of innate sensing was HCV RNA density-dependent. Data support the active contribution of cell-associated HCV in the loss-of-function of the danger signal responsiveness of a sub-population of myeloid DC *in vivo*, which might contribute to the failure of chronically infected patients to generate and maintain long-term HCV-specific CD4^+ ^T cell responses.

### Anti-viral drugs and vaccination

The current anti-HCV therapy is based on the use of polyethylene glycol modified IFN-α in combination with ribavirin. However, this treatment is expensive, relatively toxic and effective in only half of treated patients. New therapies are therefore needed. Besides the development of small anti-protease and anti-polymerase inhibitors, other approaches like siRNAs targeting HCV sequences offer potentially new approaches for therapeutic intervention as reported by **Chris Richardson **(Dalhousie University, Halifax, Canada). Although resistance can be observed in replicon cell lines in the presence of a single siRNA, using more than one siRNA targeting different regions of HCV genome can reduce the appearance of resistant mutants. A major problem with the siRNA approach is the delivery of these molecules into the cytosol. Delivery of these molecules in the form of shRNA with an adenovirus vector might be a way to circumvent this problem [56,57].

**Geneviève Inschauspé **(Transgene, Lyon, France) gave a talk on 'Hopes and beyond in the development of vaccines against hepatitis C virus' based on the view that long lasting and specific T cell responses correlate with lowering of HCV viremia [58]. Vaccines that have so far reached the clinics are not tailored to mount such responses. Second generation vaccines are being developed with the help of murine-based surrogate challenge assays providing for the *in vivo *testing of vaccine induced T cells. These assays typically use recombinant vaccinia viruses or *listeria monocytogenes *[59]. Screenings with such assays have indicated that combination of non-structural antigens (NS3 to NS5) are sufficient to induce protective immunity. A proof-of-concept study has recently been performed in the chimpanzee model confirming that a NS-based T-cell vaccine can induce non-sterile immunity in 80% of the vaccinees [60]. The first vectored vaccine based on three HCV NS antigens carried by the MVA pox virus strain is entering the clinics (Transgene).

**Mark Wainberg **(Jewish General Hospital, McGill University, Montréal, Canada) presented on differences among HIV-1 subtypes involving the prevalence of the K65R mutation in reverse transcriptase (RT). This mutation has principally been identified in the context of tenofovir usage as part of antiretroviral drug regimens in western countries, in which subtype B viruses are most predominant. However, the K65R mutation is seen relatively rarely in comparison with other substitutions, and, indeed, tenofovir (TDF)-based regimens have been shown to be durable over long periods, when this drug is used in association with two other efficient antiviral compounds, e.g. 3TC and efavirenz. Recently, the Wainberg laboratory published data showing that selection of the K65R mutation in tissue culture with subtype C viruses under tenofovir pressure led to appearance of the K65R mutation far more rapidly than occurred with viruses of subtype B origin. The reasons for this are not well understood, but may relate to differences, between viruses of subtypes B and C in coding sequences at amino acid positions 64–66 within the HIV-1 reverse transcriptase enzyme. More recently, studies of patients in Botswana, where subtype C viruses are predominant, who failed antiretroviral therapy based on use of ddI/d4T/3TC, were shown to have developed the K65R mutation with relatively high frequency. Thus, it appears that development of the K65R mutation in subtype C viruses may not be specific to the drug utilized but more to the viral subtype. In brief, subtype C viruses may have a greater preponderance to develop the K65R mutation than do viruses of subtype B. These findings highlight the need to monitor for the presence of drug resistance mutations in settings in which HIV-1 viruses other than those of subtype B are likely to predominate and to characterize drug resistance mutations associated with different viral subtypes [61-63].

**Jean-Pierre Routy **(Centre Universitaire de Santé McGill, Montréal, Canada) addressed the important topic of "The potential for HIV eradication". JP Routy's lecture dealt with the potential of valproic acid to potentially play a role in clearing latent HIV infection. The mechanism whereby this might take place could be through interaction with histone molecules in a way as to provoke latently infected cells to become overt producers of progeny viruses. This, in turn, might result in their death. JP Routy explained that differences exist among cells of lymphocytic vs monocytic lineage, and that the use of adjuvant therapies, including valproic acid, as well as that of various interleukin molecules, needs to be explored in the context of clinical trials. He explained that different cellular reservoirs might act in differential fashion when stimulated by chemicals such as valproic acid and/or a variety of interleukin compounds, such as IL-2, IL-7, and IL-10. He pointed out that a number of clinical trials to test the hypothesis that inhibitors of histone deacetylase might play important roles with respect to provoking expression of integrated proviral DNA are underway. Conceivably, the simultaneous use of agents such as valproic acid, together with effective antiretroviral compounds, could make a difference in regard to reservoir longevity [64-66].

Host cell editing of nascent retroviral cDNA has been furiously hot topic since Michael Malim's group identified APOBEC3G as a key molecule restricting HIV DNA replication on a Δ*vif *background [67]. **Simon Wain-Hobson **(Institut Pasteur, Paris, France) spoke on mechanistic parallels between cytidine and adenosine deamination of viral nucleic acids. The APOBEC3G enzyme deaminates multiple cytidine residues to yield uracil residues effectively mutating the genome to death. In experimental settings HBV, HTLV-I and foamy viruses are vulnerable to editing by this enzyme and others of this 8 gene family. *In vivo *editing occurs for HBV and follows up-regulation of APOBEC3G by type I interferons. While a growth industry, genetic editing of viruses has been known for more than 20 years. The prototype is adenosine deamination of double stranded RNA of measles virus by the type I interferon induced ADAR-1L enzyme. Indeed ADAR-1L and APOBEC3 molecules harbour a highly conserved HXEXnCXXC motif in the crucial zinc finger. These similarities suggest an ancient gene duplication and evolution into paralogs with different substrate specificities [68-70].

On the topic of the mechanisms of resistance to antiretroviral drugs, **Vincent Calvez **(Hôpital de la Pitié-Salpetrière, Paris, France) discussed the mechanisms of resistance to HAART that are very complex and important issues for people living with HIV. As pointed out by V. Calvez, HIV-1 exemplifies the principles of Darwinian evolution because of its high replication and mutation rates. This allows a close look at evolution within periods of days. Certain selective pressures that drive the evolution of HIV include chemotherapy, anatomic compartmentalization and the immune responses. Resistance to HAART, and in particular the increasing levels of transmitted resistant viral strains could offset the substantial gains won with potent antiretroviral therapy. Primary and acquired antiretroviral resistance rates reflect the relative usage of different antiretroviral drugs in the population living with HIV, as well as the inherent genetic barrier to the development of resistance associated with individual drugs. Data on antiretroviral resistance rates, gleaned from the growing HIV-1-infected population treated with a continuously increasing number of antiretroviral drug combinations, provide insights into patient management approaches for delaying the emergence of resistance and minimizing the degree of resistance. Evolving data suggest that the relative ease with which HIV-1 escapes the selective pressure of chronic drug exposure varies for the different antiretroviral drug classes and individual antiretroviral drugs. The development of resistance *in vivo *can be anticipated based on these data, in conjunction with the individual's treatment history and resistance testing results. These in turn can guide clinicians to adapt HAART treatments and to preserve therapeutic options for the time when antiretroviral-resistant strains emerge. The recent developments of new antiretroviral drugs, such as anti-integrase and boosted protease inhibitors, suggest that several treatment strategies can limit the development of resistance.

### Prevention

**Julie Bruneau **(Centre de Recherche du CHUM, Université de Montréal, Canada) focused her presentation on harm reduction strategies to prevent the transmission of HIV and HCV among injection drug users. Studies in a variety of countries provided evidence that Needle Exchange Programs (NEP) can reduce the incidence of HIV infection among injection drug users (IDU) [71-75]. In contrast, studies in Montréal and Vancouver attracted international attention upon reporting an independent association between NEP attendance and HIV seroconversion [76,77]. These inconsistent findings indicate a need for more informative assessments of the underlying conditions influencing the impact of NEPs. J. Bruneau presented a study examining patterns of utilisation of syringe access programs and geographic proximity to the IDU' dwelling place, in relation to high-risk syringe sharing behaviours in a population of IDUs living in Montréal. Her data confirms that NEPs were implemented in areas of high IDU density, and demonstrates a positive impact of a NEP fixed site implemented in such an environment on HIV prevention. This relationship could be modified, however, by other attributes of neighbourhoods and is not as straightforward as suggested by previous reports [78,79]. In addition, she reported results suggesting that regardless of the distance, IDUs who consistently acquire their syringes from the same source are less than half as likely to report high-risk injection behaviour.

### Future pandemics by influenzae viruses

**Bruno LINA **(HCL, Université de Lyon, France) gave an overview on new influenza viruses, such as H5N1, as infectious agents responsible for respiratory tract infections. For entry human influenza viruses bind sialic acids (SA) that are linked to glycans via an α2–6 linkage, while avian influenzae better recognize α2–3 linked SA. This difference can partly explain why avian viruses do not infect humans. Until recently, it was postulated that no α2–3 linked SA were present in man, because the upper respiratory tract cells harbour only α2–6 linked sialic acids. The description of H5N1 human cases led to a closer look at SA linkage in the lower respiratory tract, showing that ciliated airway and type II alveolar epithelial cells could display α2–3 linked sialic acids while α2–6 SA was detected in ciliated and gobelet cells. In the haemagglutinin pocket of the receptor-binding site, several amino acids are key elements of the α2–3 versus α2–6 specificity. For the well-known 1918 influenza A pandemic strain, it has been suggested that one single avian to human substitution has been responsible for the adaptation of this deadly emerging virus. Although the distribution of specific receptors on target organs and specific amino acid signatures are major factors in the host range restriction of influenza A viruses, other parameters are controlling influenza replication in cells. For example, it has been shown that the replicase complex of the avian viruses is not efficient in the context of a human cell, because human cells lack the protein counterparts present in the avian cell. Therefore, as pointed out by B. Lina, although necessary for the emergence of influenza pandemic viruses, the adaptation of the virus to its new receptor is probably not the only key modification necessary for the emergence of a new pandemic.
